# Side Chain Effect of Hydroxypropyl Cellulose Derivatives on Reflection Properties

**DOI:** 10.3390/polym11101696

**Published:** 2019-10-16

**Authors:** Kenichiro Hayata, Seiichi Furumi

**Affiliations:** 1 Department of Chemistry, Graduate School of Science, Tokyo University of Science, 1-3 Kagurazaka, Shinjuku, Tokyo 162-8601, Japan; 1319599@ed.tus.ac.jp; 2 Department of Applied Chemistry, Faculty of Science, Tokyo University of Science, 1-3 Kagurazaka, Shinjuku, Tokyo 162-8601, Japan

**Keywords:** cellulose, cholesteric liquid crystal, Bragg reflection, thermotropic liquid crystals, ester, carbamate

## Abstract

Some cellulose derivatives are known to exhibit thermotropic and lyotropic cholesteric liquid crystal (CLC) phases with a visible reflection feature by changing the side chains and mixing with specific solvents, respectively. Although many studies have been reported so far, most of the derivatives have the side chains of linear alkyl groups, but not the bulky phenyl groups. In this report, we synthesized a series of hydroxypropyl cellulose (HPC) derivatives that possessed both linear propionyl esters and bulky (trifluoromethyl)phenyl carbamates in the side chains. The reflection peaks of HPC derivatives shifted to longer wavelengths upon heating due to an increase in the CLC helical pitch. Such thermally induced shifting behavior of the reflection peak was crucially dependent on not only the propionyl esterification degree, but also the substituents in the side chains of HPC derivatives. When the side chains of HPC were chemically modified with both propionyl esters and bulky substituents such as 3,5-bis(trifluoromethyl)phenyl carbamates, the reflection peaks emerged at longer wavelengths at the same temperature. This probably happened because of the steric hindrance of bulky side chains, as supported by the empirical molecular modeling calculation. Although the occupied volumes of (trifluoromethyl)phenyl groups were independent of the CLC phase temperature with visible Bragg reflection, the substituent position, i.e., substituent orientation of trifluoromethyl groups affected the CLC phase temperature. Moreover, we found that the hydrogen bonds between carbamate moieties in the HPC side chains play an important role in the thermally induced shift of reflection peaks.

## 1. Introduction

Cholesteric liquid crystal (CLC) compounds show parallel aligned nematic liquid crystal layers of chiral molecules, resulting in the formation of periodically helicoidal supramolecular structures by accumulating the nematic liquid crystal layers in a clockwise or counterclockwise manner [1,2,3]. One of their unique characteristics is selective light reflection, the so-called Bragg reflection. The maximum reflection wavelength (*λ*) is numerically determined by the average refractive index (*n*) and helical pitch length (*p*) of the CLC compounds, which can be expressed by the following equation [3,4]:

(1) λ = np

One of the cellulose derivatives, hydroxypropyl cellulose (HPC), is known to exhibit thermotropic CLC phase with visible Bragg reflection by appropriate chemical modification of the side chains [5,6,7,8,9]. In previous reports on the optical properties of cellulose derivatives, the reflection wavelength was controlled by changing the side chain length and modification rates in the thermotropic CLCs, and concentrations in the lyotropic CLCs [10,11,12,13,14,15,16,17,18,19]. While most of them have one or two linear alkyl groups in the side chains, very few studies have reported on the CLCs from the derivatives that possess bulky substituents such as phenyl groups [20]. In this study, we synthesized a series of HPC derivatives with side chains of both linear alkyl and bulky phenyl groups. As will be seen below, we elucidated the relationships between the optical properties and occupied volumes of side chains using the empirical molecular modeling calculation. Moreover, we made our argument based on the slope coefficients of reflection wavelength against temperature, as compared with our previous results [18]. This report could contribute to the development of novel functional HPC derivatives tethering fluorescent and photochromic dyes in the side chains.

## 2. Experimental Section

**Figure 1:**
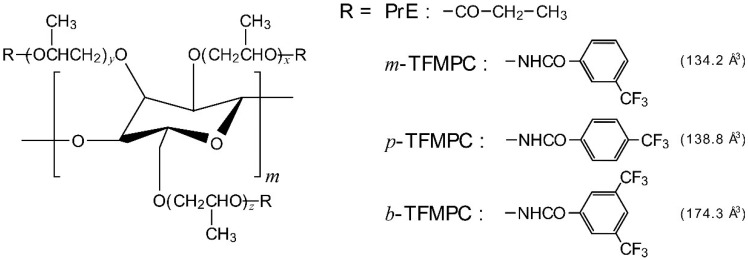
Chemical structures of hydroxypropyl cellulose (HPC) derivatives that possess both propionyl esters and three kinds of (trifluoromethyl)phenyl carbamates in the side chains. The numbers on the right side of the (trifluoromethyl)phenyl groups are their molecular occupied volumes estimated by the empirical molecular modeling simulation.

Figure 1 shows the chemical structures of HPC derivatives and their side chains. We calculated the occupied volumes of three kinds of bulky phenyl groups such as 3-(trifluoromethyl)phenyl, 4-(trifluoromethyl)phenyl, and 3,5-bis(trifluoromethyl)phenyl groups by using the empirical molecular modeling simulation (Accelrys, San Diego, CA, USA, Material Studio), as given in this figure.

We used hydroxypropyl cellulose (FUJIFILM Wako Pure Chemical Industries, Ltd., Osaka, Japan) as the starting material (Hydroxypropyl cellulose 2.0~2.9; Weight average molecular weight; *M_w_* = 2.8 × 10^4^; Number average molecular weight; *M_n_* = 1.1 × 10^5^). An aqueous solution of this HPC with the concentration of 2.0 wt% showed a viscosity of 2.0~2.9 mP·s measured at 20 °C. HPC was dried in vacuo at room temperature over 12 hours prior to use. The average number of combined propylene oxide per anhydroglucose unit (Molecular substitution; *MS*) was evaluated from the ^1^H-NMR spectral result of pristine HPC in CDCl_3_ [21]. We estimated an *MS* value of 4.0, meaning the sum of *x*, *y*, and *z* values depicted in Figure 1. Additionally, we determined the average number of hydroxy groups substituted per anhydroglucose unit (Degree of substitution; *DS*) in HPC as 2.4 by titration with trichloroacetyl isocyanate and estimation from the changes in ^1^H-NMR spectrum. The details of the procedure are described in a previous report [21]. As acyl chloride and isocyanate derivatives reacted with HPC, we adopted propionyl chloride (Tokyo Chemical Industry Co., Ltd., Tokyo, Japan), 3-(trifluoromethyl)phenyl isocyanate (Tokyo Chemical Industry Co., Ltd., Tokyo, Japan), 4-(trifluoromethyl)phenyl isocyanate (Tokyo Chemical Industry Co., Ltd. Tokyo, Japan) and 3,5-bis(trifluoromethyl)phenyl isocyanate (Tokyo Chemical Industry Co., Ltd., Tokyo, Japan). These reagents were used as received.

We synthesized an HPC propionyl ester (HPC-PrE) by esterification with propionyl chloride according to our previous report [14]. A series of HPC derivatives with different substituents in the side chains were synthesized by a two-step pathway of chemical reactions. Briefly, HPC (1.0 eq) was completely dissolved in acetone at room temperature, and subsequently pyridine (2.0 eq) was added. After heating at 55 °C, the corresponding (trifluoromethyl)phenyl isocyanate derivative (0.3 eq) was added into the reaction solution. After the reaction proceeded for 4 hours, propionyl chloride (1.3 eq) was added. Then, the reaction was continued for another 20 hours. The reaction solution was placed dropwise into a large amount of ultra-pure water for purification. The resultant viscous polymer obtained from water was dissolved again in acetone, and dropped into water in the repeated manner. The purified HPC derivatives were dried in vacuo at room temperature. In this way, we obtained three kinds of HPC derivatives with both propionyl esters and (trifluoromethyl)phenyl carbamates, which were abbreviated herein as HPC-PrE/*m*-TFMPC, HPC-PrE/*p*-TFMPC, and HPC-PrE/*b*-TFMPC.

In order to measure the transmission spectral changes upon heating process, we fabricated CLC cells according to a conventional procedure, as mentioned in our previous report [18]. A glass substrate was spin-coated by an aqueous solution of poly(vinyl alcohol) (PVA), and was then dried in an oven heated at 100 °C. After that, the PVA surface was uniaxially rubbed for generation of well-aligned CLCs. Finally, the HPC derivatives were sandwiched between a pair of the glass substrates with polytetrafluoroethylene film spacers with a thickness of approximately 200 μm.

## 3. Results and Discussion

### 3.1. Syntheses of HPC Derivatives

Firstly, we compared FT-IR spectra of the pristine HPC and its derivatives. The pristine HPC showed a broad band in a range of wavenumbers from 3000 cm^−1^ to 3600 cm^−1^ attributed to O-H stretching vibration of the terminal hydroxy groups. When HPC was reacted with propionyl chloride, 3-(trifluoromethyl)phenyl isocyanate, 4-(trifluoromethyl)phenyl isocyanate or 3,5-bis(trifluoromethyl)phenyl isocyanate, the broad band in the FT-IR spectrum absolutely disappeared, and a sharp peak concurrently appeared at 1700 cm^−1^, which was attributed to C=O stretching vibration of the ester or carbamate groups. This result implies that the terminal hydroxy groups of HPC are completely modified.

Subsequently, the numbers of substituents by propionyl esters and (trifluoromethyl)phenyl carbamates were quantitatively evaluated from the ^1^H-NMR spectral results. The degrees of modification of propionyl esters and (trifluoromethyl)phenyl carbamates were defined here as *PrE* and *TFMPC*, respectively. According to the calculation procedure in our previous report [14], the degrees were evaluated as *PrE* = 2.98 in HPC-PrE, and *PrE*:*b-TFMPC* = 2.71:0.29 in HPC-PrE/*b*-TFMPC. We synthesized HPC-PrE/*m*-TFMPC and HPC-PrE/*p*-TFMPC with different modification degrees of the side chains. The degrees were evaluated as *PrE*:*m-TFMPC* = 2.62:0.31 and 2.36:0.56 in two kinds of HPC-PrE/*m*-TFMPC and *PrE*:*p-TFMPC* = 2.59:0.32 and 2.49:0.48 in two kinds of HPC-PrE/*p*-TFMPC. In our preliminary experiment, we found that the thermotropic CLC phase does not appear for the HPC derivatives with high *TFMPC* values, such as approximately 1.0.

### 3.2. Reflection Properties of Thermotropic HPC Derivatives

**Figure 2:**
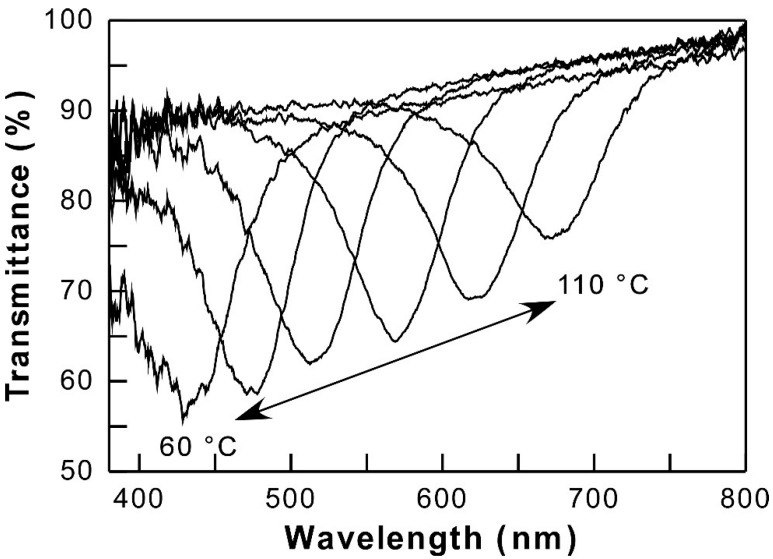
Changes in the transmission spectrum of an HPC derivative with both propionyl esters and *p*-(trifluoromethyl)phenyl carbamates (HPC-PrE/*p*-TFMPC; *PrE*:*p-TFMPC* = 2.59:0.32) as a function of temperature.

Figure 2 shows the representative results of transmission spectral changes of HPC-PrE/*p*-TFMPC as a function of temperature. Although a CLC cell of HPC-PrE/*p*-TFMPC showed optical transparency at room temperature, a Bragg reflection peak around 400 nm was observed as heating at 60 °C. By elevating the temperature, the reflection peaks gradually shifted to the longer wavelength due to an increase in the CLC helical pitch according to Equation (1). When the CLC cell was heated over 110 °C, the peak disappeared entirely.

**Figure 3:**
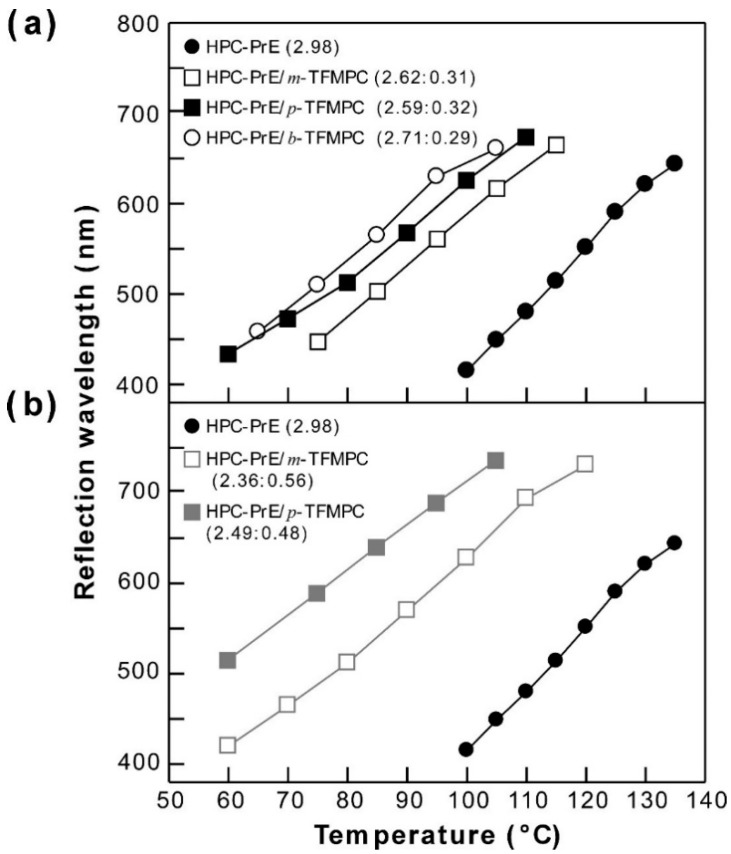
Temperature dependences of Bragg reflection wavelengths observed from a series of HPC derivatives with propionyl esters and (trifluoromethyl)phenyl carbamates. In the profiles of (**a**) and (**b**), the (trifluoromethyl)phenyl carbamate degrees were 0.29~0.32 and 0.48~0.56, respectively.

Figure 3a shows the temperature dependence of Bragg reflection wavelengths observed from CLC cells of HPC-PrE, HPC-PrE/*m*-TFMPC, HPC-PrE/*p*-TFMPC, and HPC-PrE/*b*-TFMPC. In the case of HPC-PrE, we observed the visible reflection as heating over 110 °C (Figure S1, Supplementary Materials). On the other hand, HPC-PrE/*m*-TFMPC, HPC-PrE/*p*-TFMPC, and HPC-PrE/*b*-TFMPC exhibited visible Bragg reflection peaks at temperatures lower by ~40 °C than HPC-PrE (Figures S2 and S3, Supplementary Materials). In other words, when we observed at the same temperature, the reflection peaks of HPC-PrE/*m*-TFMPC, HPC-PrE/*p*-TFMPC, and HPC-PrE/*b*-TFMPC appeared at longer wavelengths, as compared to that of HPC-PrE. This was probably due to the expansion of the CLC helical pitch caused by tethering the bulky substituents such as 3-(trifluoromethyl)phenyl, 4-(trifluoromethyl)phenyl, and 3,5-bis(trifluoromethyl)phenyl groups in the side chains of HPC derivatives.

In order to confirm the effect of the substituent position, i.e., the substituent orientation of trifluoromethyl group, we compared the experimental results of HPC-PrE/*m*-TFMPC and HPC-PrE/*p*-TFMPC. HPC-PrE/*p*-TFMPC exhibited a visible reflection peak at a lower temperature than HPC-PrE/*m*-TFMPC. Therefore, we estimated the occupied volumes of 3-(trifluoromethyl)phenyl and 4-(trifluoromethyl)phenyl groups, i.e., the positional isomers of the (trifluoromethyl)phenyl group, by using the empirical molecular modeling simulation. The values are noted in Figure 1. As a result, it was turned out that the occupied volume of the 4-(trifluoromethyl)phenyl group is slightly larger than that of the 3-(trifluoromethyl)phenyl group. Considering the results, we prepared HPC-PrE/*b*-TFMPC whose 3,5-bis(trifluoromethyl)phenyl group has two trifluoromethyl moieties at both *m*-positions. By the empirical molecular calculation, its occupied volume was estimated to be 174.3 Å^3^. Therefore, we anticipated that HPC-PrE/*b*-TFMPC would exhibit Bragg reflection in the visible wavelength at much lower temperatures than HPC-PrE/*p*-TFMPC and HPC-PrE/*m*-TFMPC. However, contrary to our expectations, the behavior of HPC-PrE/*b*-TFMPC upon heating was very similar to that of HPC-PrE/*p*-TFMPC.

This situation motivated us to gain more insight into the above-mentioned results. We prepared HPC-PrE/*m*-TFMPC and HPC-PrE/*p*-TFMPC with higher modification degrees of *m-TFMPC* and *p-TFMPC*, respectively. Figure 3b shows the reflection properties of HPC-PrE/*m*-TFMPC (*PrE*:*m-TFMPC* = 2.36:0.56) and HPC-PrE/*p*-TFMPC (*PrE*:*p-TFMPC* = 2.49:0.48). At the high *TFMPC* degree of ~0.5, HPC-PrE/*b*-TFMPC did not clearly show the reflection peak attributed to the CLC phase. Although the reflection peaks of HPC-PrE/*m*-TFMPC and HPC-PrE/*p*-TFMPC with *TFMPC* degrees of ~0.3 appeared at a longer wavelength rather than HPC-PrE at the same temperature, we found that the differences between their profiles become more obvious. This indicates that the content of the 4-(trifluoromethyl)phenyl group in HPC-PrE/*p*-TFMPC makes a larger contribution to the expansion of CLC helical pitch than that of the 3-(trifluoromethyl)phenyl group in HPC-PrE/*m*-TFMPC. The molecular lengths from carbamate to the trifluoromethyl group were different from the *m*- or *p*-positions of the substitutent. Therefore, it is plausible that the HPC derivatives possessing 4-(trifluoromethyl)phenyl (HPC-PrE/*p*-TFMPC) or 3,5-bis(trifluoromethyl)phenyl groups (HPC-PrE/*b*-TFMPC) exhibits Bragg reflection in a visible wavelength range at lower temperature, as compared with the HPC derivative with 3-(trifluoromethyl)phenyl groups (HPC-PrE/*m*-TFMPC). Furthermore, HPC-PrE/*b*-TFMPC showed a broad reflection band in a slightly narrow temperature range between 65 °C and 105 °C due to the disturbance of the helicoidal supramolecular structure at CLC phase by a large-occupied volume and the branched chemical structure of the 3,5-bis(trifluoromethyl)phenyl group. Consequently, we can envisage that the occupied volumes of side chains of HPC derivatives are independent of the CLC phase temperature with visible reflection, but are related to the stability of the helicoidal supramolecular structure of CLC. On the contrary, the substituent position of trifluoromethyl groups would affect the CLC phase temperature.

Finally, we analyzed the slope coefficients (*k*) in shifting profiles of reflection peaks from 400 nm to 600 nm as a function of temperature by the least squares method, as compiled in Table 1 [18]. The small values of *k* with a unit of nm/°C represent a small wavelength fluctuation in the thermally induced shift of reflection peak and vice versa. As is evident from the *k* values in Table 1, HPC-PrE/*m*-TFMPC, HPC-PrE/*p*-TFMPC, and HPC-PrE/*b*-TFMPC with *TFMPC* degrees of ~0.3 or ~0.5 have smaller *k* values than HPC-PrE. This is probably because the thermally induced expansion of the CLC helical pitch is hampered by hydrogen bonds in carbamate groups. As shown in Figure 2, when HPC-PrE/*p*-TFMPC (2.59:0.32) was heated at 60 °C, the reflection spectrum contained noises below 450 nm. Therefore, we recalculated the *k* value from the reflection peaks of HPC-PrE/*p*-TFMPC (2.59:0.32) between 80 °C and 100 °C, excluding 60 °C and 70 °C. As a result, the *k* value was estimated to be 5.65, which almost corresponds to the *k* values of HPC-PrE/*m*-TFMPC (2.62:0.31) and HPC-PrE/*b*-TFMPC (2.71:0.29). Furthermore, we found the smallest *k* values for HPC derivatives with *TFMPC* = ~0.5 rather than those of *TFMPC* = ~0.3. At the high *TFMPC* degrees of ~0.5, HPC-PrE/*p*-TFMPC showed smaller *k* values compared with HPC-PrE/*m*-TFMPC, probably owing to the substituent position of the trifluoromethyl group. By considering the overall results, we investigated that high degrees of modification of carbamate groups result in the smaller *k* values due to the formation of stable helical molecular structures at the CLC phase by their hydrogen bonds. Moreover, we recently found similar *k* values of ~5.9 for HPC derivatives possessing both propionyl esters and alkyl carbamates at almost the same modification degrees [18]. This suggests that the *k* value degree might be governed by hydrogen bonds between carbamate groups, but not by the steric hindrance of (trifluoromethyl)phenyl groups. Especially, in the case of a high *TFMPC* value of ~0.5, we found the that the substituent position of trifluoromethyl group has the effect on *k* value.

## 4. Conclusions

We described the reflection properties of HPC derivatives that possess different side chains of both linear propionyl esters and bulky (trifluoromethyl)phenyl carbamates. Considering the occupied volumes calculated by the empirical molecular modeling simulation, we adopted three kinds of (trifluoromethyl)phenyl groups. Some of the HPC derivatives exhibited visible Bragg reflection peaks at relatively low CLC phase temperatures, whereas the CLC phase temperatures were not dependent on the occupied volumes of side chains. When pristine HPC was chemically modified with both propionyl esters and (trifluoromethyl)phenyl carbamates in the side chains, the temperature ranges with visible reflection were wide compared to that of the HPC derivative with solely propionyl esters. We investigated that the hydrogen bonds between carbamate moieties in the HPC side chains and substituent position effect of trifluoromethyl group have a remarkable influence on the thermally induced expansion of CLC helical pitch, i.e., the reflection peak shift range, rather than the steric hindrance in side chains. The present report includes promising guidelines for the rational molecular designs and syntheses for novel CLC materials from environment- and human-friendly biomasses such as cellulose.

## Figures and Tables

**Table 1:** The slope coefficient (*k*) values of HPC derivatives in the shifting profiles of reflection peaks as a function of temperature.

HPC Derivatives	Modification Degrees (*PrE:TFMPC*)	*k* (nm/°C)
HPC-PrE	2.98:0	6.96
HPC-PrE/*m*-TFMPC	2.62:0.31	5.70
HPC-PrE/*m*-TFMPC	2.36:0.56	5.21
HPC-PrE/*p*-TFMPC	2.59:0.32	4.76 (5.65) *
HPC-PrE/*p*-TFMPC	2.49:0.48	4.99
HPC-PrE/*b*-TFMPC	2.71:0.29	5.67

* At first, the *k* value of HPC-PrE/*p*-TFMPC (2.59:0.32) could not be correctly analyzed due to the noise in the transmission spectrum heated at 60 °C. Therefore, we recalculated the *k* value using the reflection peaks at the temperature range between 80 °C and 100 °C, excluding 60 °C and 70 °C.

## Supplementary Materials

The following are available online at https://www.mdpi.com/2073-4360/11/10/1696/s1, Figure S1: Transmission spectral changes of HPC-PrE as a function of temperature, Figure S2: Transmission spectral changes of HPC-PrE/*m*-TFMPC as a function of temperature, Figure S3: Transmission spectral changes of HPC-PrE/*b*-TFMPC as a function of temperature.
