# Supplementary material for: Side Chain Effect of Hydroxypropyl Cellulose Derivatives on Reflection Properties
Source: Polymers (Basel). 2019 Oct 16;11(10):1696. doi: 10.3390/polym11101696 (PMC6835709; doi:10.3390/polym11101696)
Supplement: Supplementary file 1 [file polymers-11-01696-s001.pdf]

Submitted to *Polymers* as a Communication

Supplementary Materials

Side Chain Effect of Hydroxypropyl Cellulose Derivatives on Reflection Properties

Kenichiro Hayata and Seiichi Furumi*

Department of Applied Chemistry, Faculty of Science,
Tokyo University of Science
1–3 Kagurazaka, Shinjuku, Tokyo 162–8601, Japan
*Correspondence: furumi@rs.tus.ac.jp.

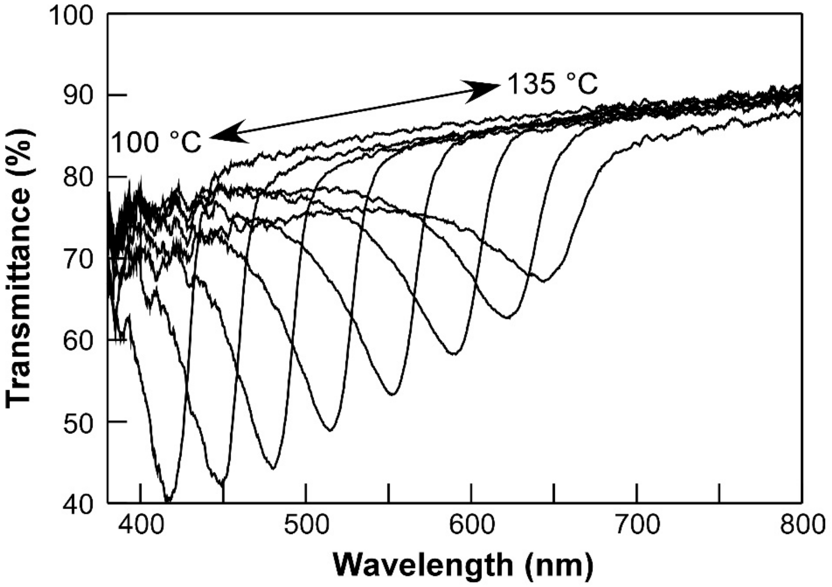

Figure S1. Changes in transmission spectrum of an HPC derivative with solely propionyl esters in the side chains (HPC-PrE; *PrE* = 2.98) as a function of temperature.

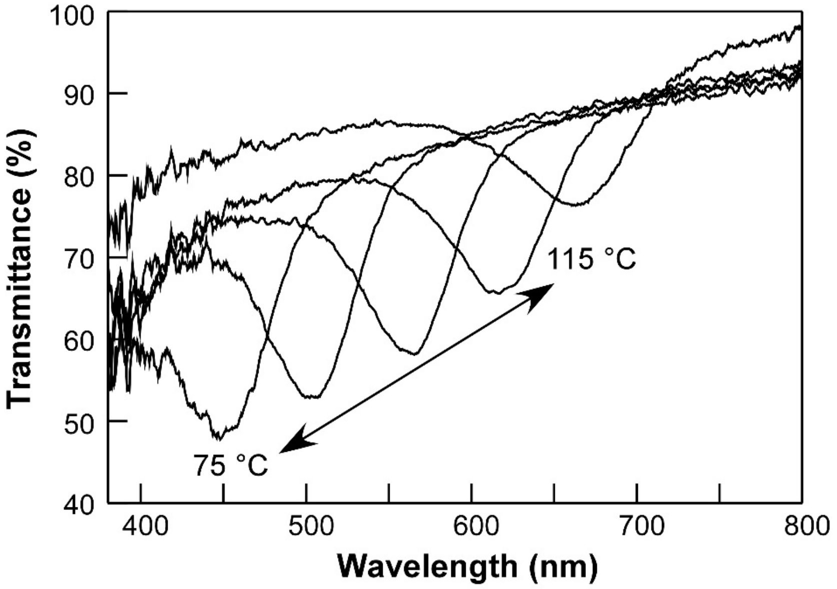

Figure S2. Changes in transmission spectrum of an HPC derivative with both propionyl esters and *m*-(trifluoromethyl)phenyl carbamates in the side chains (HPC-PrE/*m*-TFMPC; *PrE*:*m-TFMPC* = 2.62:0.31) as a function of temperature.

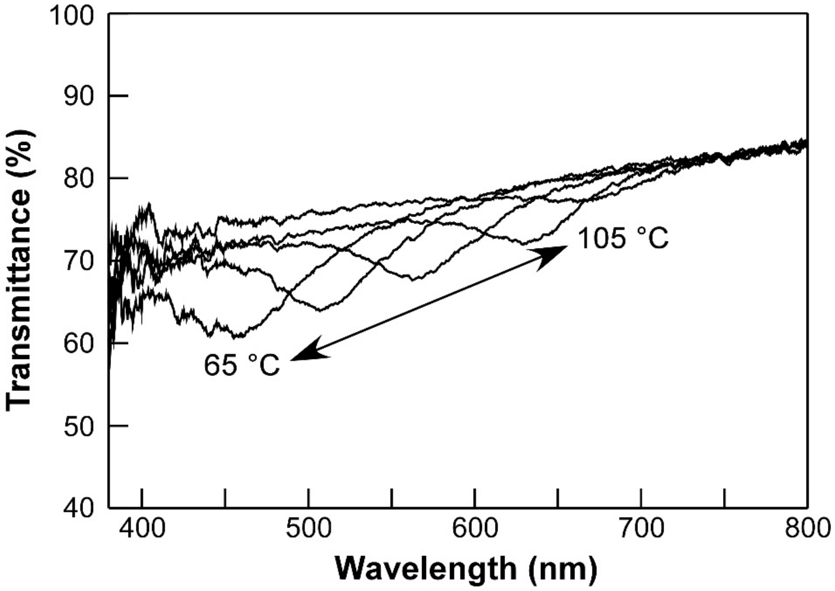

Figure S3. Changes in transmission spectrum of an HPC derivative with both propionyl esters and 3,5-*bis*(trifluoromethyl)phenyl carbamates in the side chains (HPC-PrE/*b*-TFMPC; *PrE*:*b-TFMPC* = 2.71:0.29) as a function of temperature.
